# A novel assay provides insight into tRNA^Phe^ retrograde nuclear import and re-export in *S. cerevisiae*

**DOI:** 10.1093/nar/gkaa879

**Published:** 2020-10-19

**Authors:** Regina T Nostramo, Anita K Hopper

**Affiliations:** Department of Molecular Genetics Center for RNA Biology The Ohio State University, Columbus, OH 43210, USA; Department of Molecular Genetics Center for RNA Biology The Ohio State University, Columbus, OH 43210, USA

## Abstract

In eukaryotes, tRNAs are transcribed in the nucleus and subsequently exported to the cytoplasm where they serve as essential adaptor molecules in translation. However, tRNAs can be returned to the nucleus by the evolutionarily conserved process called tRNA retrograde nuclear import, before relocalization back to the cytoplasm via a nuclear re-export step. Several important functions of these latter two trafficking events have been identified, yet the pathways are largely unknown. Therefore, we developed an assay in *Saccharomyces cerevisiae* to identify proteins mediating tRNA retrograde nuclear import and re-export using the unique wybutosine modification of mature tRNA^Phe^. Our hydrochloric acid/aniline assay revealed that the karyopherin Mtr10 mediates retrograde import of tRNA^Phe^, constitutively and in response to amino acid deprivation, whereas the Hsp70 protein Ssa2 mediates import specifically in the latter. Furthermore, tRNA^Phe^ is re-exported by Crm1 and Mex67, but not by the canonical tRNA exporters Los1 or Msn5. These findings indicate that the re-export process occurs in a tRNA family-specific manner. Together, this assay provides insights into the pathways for tRNA^Phe^ retrograde import and re-export and is a tool that can be used on a genome-wide level to identify additional gene products involved in these tRNA trafficking events.

## INTRODUCTION

tRNAs are essential adaptor molecules that deliver amino acids to the ribosome during protein synthesis. In addition to this canonical role, they also serve a host of other functions, including regulation of apoptosis and protein degradation (1). As a result, defects in the maturation of tRNAs can lead to numerous diseases in humans, such as metabolic disorders, neuromuscular diseases and cancer (2–4).

The process that generates mature, functional tRNAs from their precursor form is extensive and requires numerous gene products. In *Saccharomyces cerevisiae*, for example, >1% of the genome is dedicated to tRNA maturation (5). This maturation process can be divided into two categories. The first involves processing of the tRNA body. This includes events such as 5′ and 3′ end processing, CCA addition, nucleoside modifications, and for some tRNAs, intron removal.

The second category of tRNA maturation involves its movement within the cell (6). For many of the aforementioned tRNA processing events to take place, a tRNA needs to be in the right subcellular compartment at the appropriate time. For some tRNAs, this requires that they are trafficked bidirectionally between the nucleus and the cytoplasm to access the enzymes required for various maturation steps. Three tRNA subcellular trafficking events between the nucleus and cytoplasm have been documented in yeast. The first event is primary nuclear export, where newly transcribed end-processed tRNAs are localized to the cytoplasm (7–12). Once in the cytoplasm, tRNAs containing introns are spliced by the splicing endonuclease (SEN) complex on the surface of the mitochondria (13–16). Second, tRNAs can undergo retrograde nuclear import (17–19). This process serves several important functions, such as maturation of select tRNA species (20,21), tRNA quality control (22), translation regulation (23) and response to cellular stress (24,25). The tRNA import pathway is also hijacked by viruses such as HIV-1 for their own nuclear import (19). Lastly, in the third tRNA trafficking event, called nuclear re-export, nuclear tRNAs that were previously cytoplasmic are returned back to the cytoplasm (26).

Since translation takes place in the cytoplasm, all tRNAs must undergo primary tRNA nuclear export to access the protein synthesis machinery. However, for most tRNAs in *S. cerevisiae* the latter two trafficking events appear not to be required for the generation of a mature tRNA. As described in detail later, an exception to this is tRNA^Phe^. In response to various stress conditions, at least seven other tRNA families in yeast, such as tRNA^Tyr^ and tRNA^Pro^_UGG_, have been shown to re-enter the nucleus (18,27). Retrograde import of certain tRNA families has also been observed in human cells in response to oxidative stress (25). Given the long half-life of tRNA molecules and the many roles of the retrograde import process in maintaining proper cellular functioning, it would not be surprising if most, if not all, families of tRNAs undergo retrograde nuclear import and re-export at least once in their lifetime. Therefore, understanding how tRNAs are escorted between the nucleus and cytoplasm will provide important insights into cell biology and pathobiology.

In order to identify the gene products involved in tRNA biogenesis and trafficking, our lab previously designed a genome-wide Northern blot assay to screen the *S. cerevisiae* deletion and temperature-sensitive collections, using tRNA^Ile^_UAU_ as a reporter (28). In total, 162 novel gene products were identified to function in the many stages of tRNA maturation, including production of the initial transcript, 5′ and 3′ end processing, primary nuclear export, delivery of tRNAs to the mitochondrial surface and/or localization, assembly or function of the SEN complex, and intron turnover. Overall, the findings from this analysis have (and continue to) greatly enhanced our understanding of how tRNAs are processed and initially escorted from the nucleus to the cytoplasm. For example, the screen led to the discovery of three mitochondrial outer membrane proteins, Tom70, Tom22 and Sam37, which are required for splicing of pre-tRNAs on the surface of the mitochondria (16). Furthermore, the screen led to the discovery that the mRNA exporter, Mex67-Mtr2, serves as a nuclear transporter of certain tRNAs to the cytoplasm in both the primary export and re-export steps (29).

Despite the wealth of information acquired by the genome-wide screen by Wu *et al.* (28), there were a few limitations. Since the assay was dependent on assessing changes in the migration of tRNAs on polyacrylamide gels as they were undergoing various processing steps, it lacked the ability to detect gene products that regulate tRNA trafficking after a tRNA was spliced, specifically the retrograde nuclear import and re-export steps. Since cytoplasmic tRNAs that are returned to the nucleus by the retrograde import process are generally not significantly altered upon nuclear import, they migrate the same on polyacrylamide gels. Therefore, to identify gene products involved in these two trafficking events and thus more fully complete the picture of tRNA biology, we developed a simple biochemical assay using hydrochloric acid (HCl) and aniline that could be utilized on a genome-wide scale in yeast. This assay takes advantage of a modification found solely on tRNA^Phe^ at position 37, called wybutosine (yW). This particular modification is uniquely suited to identify genes involved in the latter two tRNA trafficking events because for tRNA^Phe^ to be donned with yW, it must undergo both retrograde nuclear import and re-export (20).

Here, we validate the ability of the HCl/aniline assay to detect gene products involved in tRNA retrograde nuclear import and re-export. Using this assay, we confirm the role of Mtr10 and Ssa2 in the retrograde nuclear import of tRNA^Phe^ under constitutive (Mtr10) and amino acid deprivation (Mtr10 and Ssa2) conditions. Furthermore, we show that Mex67 and Crm1, but not the canonical tRNA exporters Los1 or Msn5, mediate the constitutive re-export of tRNA^Phe^, highlighting the specificity of tRNA exporters in the re-export of different tRNA families.

## MATERIALS AND METHODS

### Strains, plasmids and media

The majority of *S. cerevisiae* strains used in this study were derived from BY4741 (*MATa his3Δ leu2Δ met15Δ ura3Δ*). This includes the following strains from the *MATa* deletion collection: *tyw1Δ*, *tyw2Δ*, *tyw3Δ*, *trm7Δ*, *ssa2Δ*, *los1Δ* and *msn5Δ*. The *mtr10Δ* strain was generated by gene replacement of the *MTR10* locus with the Nat^R^ gene (18). The *los1Δ msn5Δ* strain was generated by gene replacement of the *MSN5* locus with the *hph*^R^ gene in the *los1Δ* strain (los1::KanMX4) (23). The *mex67-5* and *crm1-1* strains were obtained from the temperature-sensitive mutant collections, kindly provided by Dr C. Boone (University of Toronto) (30). The *TRM7* and *TYW3* plasmids were obtained from the yeast tiling collection (Open Biosystem) (31).

Yeast strains were grown at 23°C in synthetic complete media supplemented with 2% glucose (SCD). For amino acid deprivation experiments, cells were washed once in SCD lacking amino acids, then resuspended in the same type of media for up to 2 h, as indicated. For temperature-sensitive strains, cells were shifted to 37°C for up to 4 h, as indicated.

### Small RNA isolation

Small RNAs (tRNAs, 5S rRNA, 5.8S rRNA, and other small RNAs) were isolated as described in Wan and Hopper (16). RNA concentration was measured using the NanoDrop 2000C (kindly provided by Dr Paul Herman, OSU).

### HCl/aniline assay

To induce wybutosine base excision, 10 μg of small RNA in 48 mM HCl were incubated for 3 h at 37°C. RNA/HCl samples were neutralized in a final concentration of 0.93 mM KOH. Controls lacking HCl contained only RNA in dH_2_O. Next, HCl-treated, neutralized RNA or HCl-untreated RNA was incubated with an equal volume of 0.5 M aniline (pH 4.5) at 60°C for 20 min to induce chain scission. RNAs were diluted in dH_2_O, then precipitated with cold 100% ethanol, 3M sodium acetate (pH 5.2) and GlycoBlue Coprecipitant (Invitrogen). After overnight incubation at −80°C, RNA was pelleted by centrifugation at 15 000 × g for 20 min, washed in 70% ethanol, and dissolved in dH_2_O. See supplemental methods for more specific assay details, including the volumes used in this assay.

### Northern blot analysis and quantification

The total volume (20 μl) of HCl and aniline-treated RNA or HCl-untreated, aniline-treated RNA was mixed with an equal volume of 2x loading dye and heated at 85°C for 5 min. As described previously (28), the RNAs were separated by electrophoresis by 7 M urea–PAGE and transferred to a Hybond N^+^ membrane (Amersham). Intact, mature tRNA^Phe^ as well as the 5′ and 3′ cleaved halves of tRNA^Phe^ were detected by chemiluminescence (16) employing a UVP ChemStudio instrument from Analytic Jena using a digoxigenin-labeled probe that was 40 nt in length and complementary to 20 nts of the 3′ end of the 5′ exon and 20 nts of the 5′ end of the 3′ exon. Subsequently, all blots were reprobed with a digoxigenin-labeled probe complementary to 5S rRNA, which was used as a loading control. The sequences of all probes are listed in the supplemental methods. To quantify the levels of tRNA^Phe^ lacking the yW modification, the amount of mature tRNA^Phe^ relative to the amount of total tRNA^Phe^ (mature plus cleaved) was measured in HCl/aniline-treated samples.

### Statistical analysis

All data are expressed as mean ± SEM. Statistical analysis was performed using the GraphPad Prism 4 software, with differences analyzed by two-tailed unpaired *t* test (if comparing only two experimental groups) or one-way ANOVA followed by Tukey's Multiple Comparisons test (if comparing more than two experimental groups). A *P* value of <0.05 was considered significant. Each experiment was performed at least in triplicate.

## RESULTS

### An HCl/aniline assay detects wybutosine-modified tRNA^Phe^

**Figure 1.**
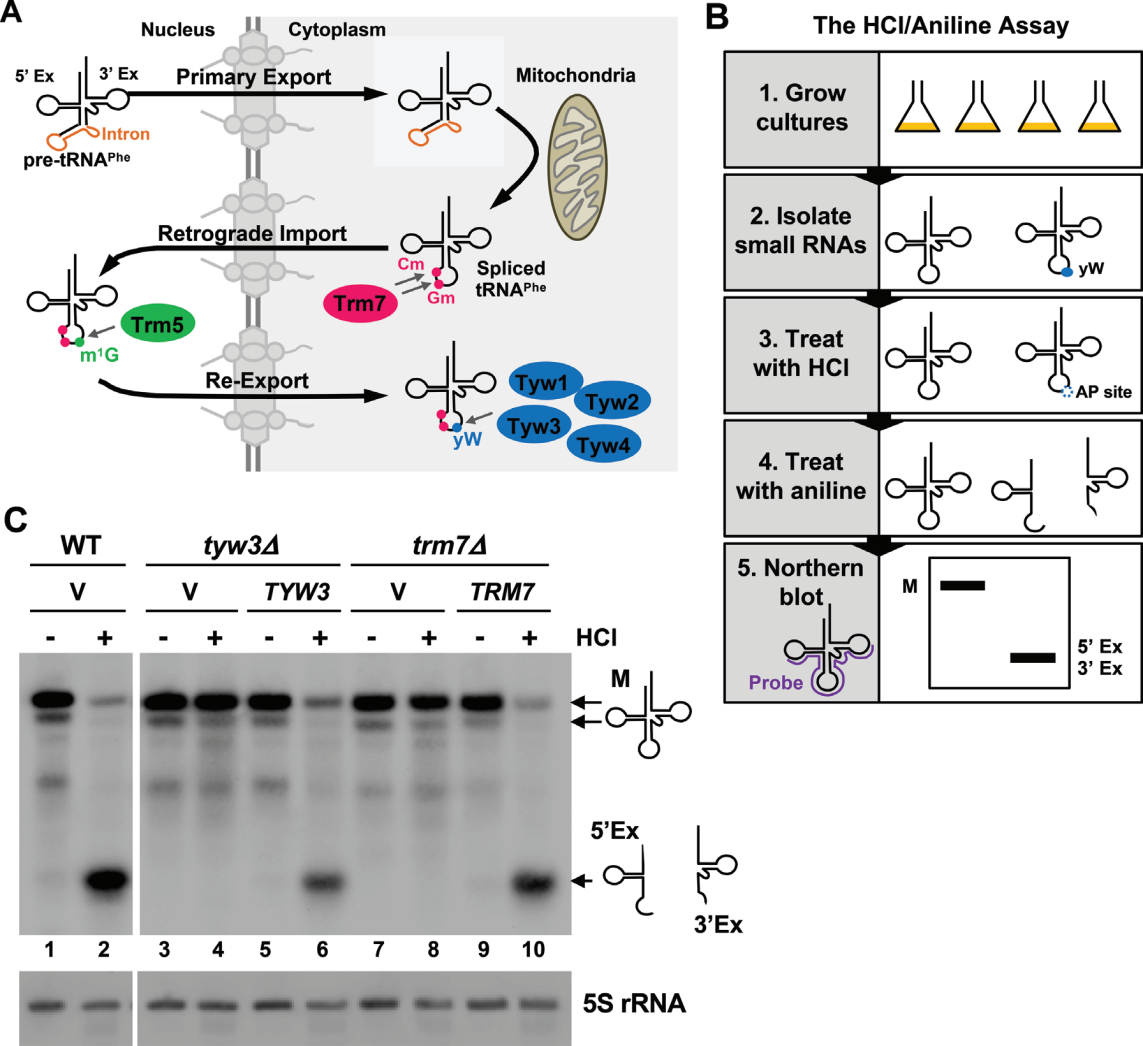
Schematic depictions and validation of an assay to detect retrograde nuclear importers and re-exporters of tRNA^Phe^ in *S. cerevisiae*. (**A**) Maturation of tRNA^Phe^ requires three trafficking events which move the tRNA back and forth between the nucleus and the cytoplasm. The primary transcript of tRNA^Phe^ is synthesized in the nucleus and undergoes 5′ and 3′ end-maturation and CCA addition (not shown), yielding precursor tRNA^Phe^ (pre-tRNA^Phe^). Pre-tRNA^Phe^ contains a 5′ and 3′ exon (black lines) separated by an intron (orange line). After decoration with numerous modifications (not shown), pre-tRNA^Phe^ is transported through the nuclear pores to the cytoplasm in a process called tRNA primary nuclear export and the intron is removed by the splicing endonuclease located on the outer surface of the mitochondria. Spliced tRNA^Phe^ is then methylated by Trm7 at positions 32 (Cm) and 34 (Gm) (shown in pink), as well as by other modification enzymes (not shown). Modified tRNA^Phe^ is trafficked back into the nucleus in a process called tRNA retrograde nuclear import, where it is now a substrate for the methyltransferase Trm5, which methylates G37 (m^1^G) (shown in green). For the final step in tRNA^Phe^ maturation, the tRNA is transported back to the cytoplasm, known as tRNA nuclear re-export, and wybutosine (yW) is added to the m^1^G in a series of reactions catalyzed sequentially by Tyw1-4 (shown in blue). (**B**) Overview of the steps in the HCl/aniline assay. Small RNAs are isolated from yeast cultures and treated with HCl. This acid treatment removes the wybutosine from tRNA^Phe^ leaving an abasic site (AP site; apurinic or apyrimidinic site). Subsequent treatment with aniline induces strand cleavage at the AP site, splitting the tRNA^Phe^ into two halves which can be visualized by Northern blot analysis using an oligonucleotide that is 40 nts in length and complementary to 20 nts on either side of the 5′/3′ exon junction, as indicated in purple in panel 5. (**C**) Validation of the HCl/aniline assay. Small RNAs isolated from wild-type (WT), *tyw3Δ* or *trm7Δ* cells expressing either a single copy vector (V) or the single copy vector containing the endogenous *TYW3* or *TRM7* gene were treated with or without HCl and subsequently exposed to aniline. Northern blot analysis was performed using the probe described in (B). The detected RNAs correspond to the mature (M), 5′ exon (5′Ex) or 3′ exon (3′Ex) of tRNA^Phe^. 5S rRNA levels serve as a loading control.

In order to identify gene products involved in the retrograde import and re-export of tRNAs, we generated an assay that exploited the unique maturation process undergone by tRNA^Phe^ (Figure 1A) (20). The pathway for tRNA^Phe^ maturation begins with processing of the primary tRNA transcript into precursor tRNA^Phe^ in the nucleus and subsequent transport to the cytoplasm. Once in the cytoplasm, the intron separating the 5′ and 3′ exons is spliced by the SEN complex, located on the surface of the mitochondria (13–16). In the cytoplasm, the spliced tRNA^Phe^ receives multiple modifications, including methylation at positions 32 (Cm_32_) and 34 (Gm_34_) by the cytoplasmic methyltransferase Trm7. Gm_34_, and to a lesser extent Cm_32_, are prerequisites for the later wybutosine modification of tRNA^Phe^ at position 37 (yW_37_) (32). The spliced tRNA^Phe^ then undergoes retrograde nuclear import, where it is methylated by Trm5 at G_37_ (m^1^G_37_) (33). Two key features of Trm5, specifically its nuclear localization and its ability to recognize only spliced tRNA^Phe^ (34,35), allow the m^1^G_37_ modification to serve as evidence that tRNA^Phe^ has undergone retrograde nuclear import following splicing on the mitochondrial surface. Finally, tRNA^Phe^ is re-exported to the cytoplasm, where yW is added to the m^1^G_37_ in a series of reactions catalyzed sequentially by the enzymes Tyw1, Tyw2, Tyw3 and Tyw4 (36). Thus, the yW modification serves as a road map of previous travel of tRNA^Phe^ bidirectionally between the nucleus and cytoplasm. By measuring levels of spliced tRNA^Phe^ that contain or lack the yW modification in yeast deletion or temperature-sensitive strains, we can identify novel gene products that modulate the retrograde nuclear import or re-export steps.

To determine whether tRNA^Phe^ contains the wybutosine modification, and thus has undergone retrograde nuclear import and re-export, we developed an assay with the potential to be used on a genome-wide scale. This simple biochemical assay, herein referred to as the hydrochloric acid (HCl)/aniline assay (Figure 1B), is based on experiments conducted in the late 1960s and 1970s (37,38) which demonstrated that mild acid treatment of tRNA^Phe-yW^ results in excision of the yW base without breaking the sugar-phosphate backbone, creating an abasic site. This abasic site is susceptible to chain cleavage via a β-elimination reaction, which can be induced by heating the tRNA in the presence of aniline at a low pH (39). Since the location of the wybutosine modification is at position 37, the final nucleotide at the 3′ end of the 5′ exon, any tRNA^Phe^ possessing the wybutosine modification would be split into its 5′ and 3′ exon fragments following sequential HCl and aniline treatment. Conversely, tRNA lacking this modification would remain intact. Mature tRNA^Phe^ and tRNA^Phe^ 5′ and 3′ exon fragments can then be visualized by non-radioactive Northern blot analysis (40).

To validate the HCl/aniline assay, we isolated small RNAs from either wild-type (WT) yeast cells or cells lacking the genes *TYW1*, *TYW2*, *TYW3* or *TRM7*, since the enzymes encoded by these genes are all required for wybutosine modification of tRNA^Phe^ (32,36). Trm5 is an additional enzyme that is required for m^1^G modification of position 37 prior to yW addition. Due to the lack of adequate *TRM5* gene deletion or temperature-sensitive strains, this gene was not used in the assay validation. Isolated RNAs were treated either with or without HCl for 3 h at 37°C to induce wybutosine base excision. This time point is sufficient to yield near complete excision of the wybutosine base (Supplementary Figure S1A). Subsequently, HCl-treated and HCl-untreated RNAs were incubated with aniline (pH 4.5) at 60°C for 20 min to induce chain scission at the abasic sites. The RNAs were purified and Northern blot analysis was performed using a 40 nt long probe that hybridizes to 20 nts of the 3′ end of the 5′ exon and 20 nts of the 5′ end of the 3′ exon (Figure 1B) (see methods).

In HCl-untreated, aniline-treated WT cells, two main tRNA^Phe^ species are evident (Figure 1C, lane 1), which correspond to mature tRNA^Phe^ (Supplementary Figure S1B, lane 1 and 2 versus 3). In *S. cerevisiae*, there are ten copies of the tRNA^Phe^ gene (41). Two of these genes differ by a single nucleotide substitution in each of the 5′ and 3′ exons. The dual gel migration pattern often observed for mature tRNA^Phe^ may be due to one or both of these substitutions, or to under-modification of a portion of the tRNA. Upon sequential treatment of RNA from WT cells with HCl and aniline, the intensity of the mature tRNA^Phe^ species is greatly reduced and a tRNA species consistent with the size of the 5′ and 3′ exons is evident (Figure 1C, lane 2; Supplementary Figure S1B, lane 2 versus. 4). The 5′ and 3′ exons of tRNA^Phe^ are 37 and 39 nucleotides in length, respectively. Under the electrophoresis conditions used in these experiments, a 40 nt long probe that hybridizes to 20 nts of the 3′ end of the 5′ exon and 20 nts of the 5′ end of the 3′ exon, reveals a single tRNA^Phe^ species upon HCl-induced yW excision and aniline-induced chain cleavage. This tRNA species corresponds to both the 5′ and 3′ exons (Supplementary Figure S1C). A second tRNA^Phe^ species of lower abundance runs slightly below this, which can be clearly detected when using a probe that hybridizes solely to the 3′ exon of tRNA^Phe^ (Supplementary Figure S1C) and is likely the result of the single nucleotide substitution in the 3′ exon in some of the tRNA^Phe^ genes (41). Greater than 90% cleavage of mature tRNA^Phe^ following HCl and aniline treatment in the WT strain (Supplementary Table S1) is consistent with previous reports that the majority of mature tRNA^Phe^ contains yW (20).

In contrast to WT cells, HCl and aniline treatment of RNAs from *tyw3Δ* and *trm7Δ* cells yields similar amounts of mature tRNA^Phe^ as HCl-untreated RNAs and does not result in the presence of tRNA^Phe^ 5′ and 3′ exon bands (Figure 1C, lanes 3 versus 4 and 7 versus 8). This indicates the lack of wybutosine modification of tRNA^Phe^, as expected. Similar findings were observed in *tyw1Δ* and *tyw2Δ* cells (Supplementary Figure S1D). Expression of a single copy plasmid containing the endogenous *TYW3* or *TRM7* gene in *tyw3Δ* and *trm7Δ* cells, respectively, restored mature tRNA^Phe^ cleavage into its 5′ and 3′ exons (Figure 1C, lanes 6 and 10). These data indicate that the absence of tRNA^Phe^ cleavage in *tyw3Δ* and *trm7Δ* cells is mediated by the *TYW3* and *TRM7* genes, respectively. The specificity of this assay for the wybutosine modification of tRNA^Phe^ was further assessed by probing for tRNA^Leu^_CAA_, which does not contain this modification. HCl/aniline treatment of RNAs from WT, *tyw1Δ*, *tyw2Δ* or *tyw3Δ* cells does not yield cleavage of mature tRNA^Leu^_CAA_ (Supplementary Figure S1D). Together, these results demonstrate that the HCl/aniline assay can specifically measure the wybutosine status of tRNA^Phe^, and thus should be able to detect tRNA retrograde nuclear importers and re-exporters.

### The HCl/aniline assay detects the tRNA^Phe^ retrograde nuclear importers Mtr10 and Ssa2

**Figure 2.**
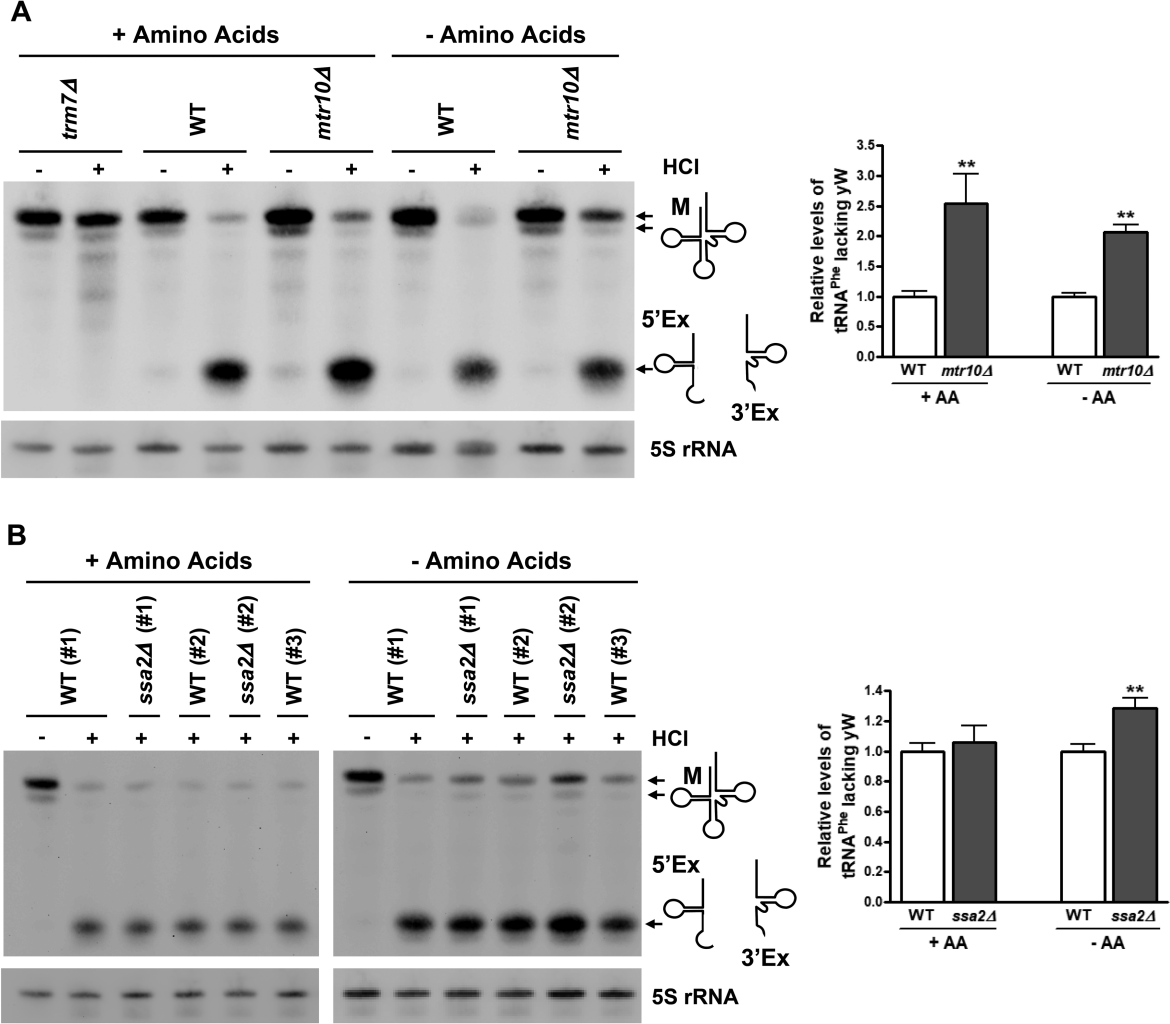
Mtr10 and Ssa2 function in constitutive and amino acid deprivation-induced retrograde nuclear import of tRNA^Phe^, respectively. Wild-type (WT), *trm7Δ*, *mtr10Δ* (**A**) and *ssa2Δ* (**B**) cells were grown in media containing (+AA) or lacking amino acids for 2 h (–AA). Small RNAs were isolated, treated with or without HCl, incubated with aniline, and visualized by Northern blot using the probe shown in Figure 1B. Representative Northern blots are shown (A and B, left). In (**B**), independent biological replicates are shown, as indicated by the number in parentheses. 5S rRNA levels serve as a loading control. The relative levels of tRNA^Phe^ lacking wybutosine (yW), shown in the graphs on the right, are calculated by dividing the amount of mature tRNA^Phe^ (M) in the +HCl sample by the total amount of tRNA^Phe^ (mature and cleaved) in that lane. For (A), *n* = 7–8 for +AA; *n* = 3 for –AA. For (B), *n* = 3 for +AA; *n* = 5–6 for –AA. All data are normalized to the WT group treated under the same nutrient conditions (which was set to 1) and are represented as mean ± SEM. **P < 0.01 relative to WT samples treated under the same nutrient conditions.

To demonstrate the feasibility of the HCl/aniline assay in the detection of putative tRNA retrograde nuclear importers, we assessed HCl/aniline-induced cleavage of tRNA^Phe^ in strains lacking either the *MTR10* or *SSA2* genes which code for proteins previously identified to play a role in the tRNA retrograde nuclear import process. Mtr10 is a member of the β-importin family that functions in the import of tRNA into the nucleus under conditions of amino acid deprivation (18). However, the role of Mtr10 in the constitutive import of tRNA is less clear. Unlike cells deficient in the tRNA re-exporter Msn5, there is a lack of nuclear accumulation of tRNA^Tyr^ in fed *msn5Δ mtr10Δ* cells. These genetic data suggest Mtr10 functions upstream of the re-export step and may play a role in constitutive tRNA import (42). In accordance with these findings, HCl and aniline treatment of RNAs isolated from *mtr10Δ* cells grown in the presence of amino acids shows that although tRNA^Phe^ cleavage products are still formed, there is an increase in the amount of uncleaved mature tRNA^Phe^ as compared to WT cells (Figure 2A). Furthermore, when *mtr10Δ* cells are grown in the absence of amino acids for 2 h, levels of uncleaved, mature tRNA^Phe^ are increased relative to amino-acid deprived WT cells.

In order to quantify these data, levels of mature tRNA^Phe^ relative to the amount of total tRNA^Phe^ (mature plus cleaved) were measured in HCl/aniline-treated samples. Since the levels of mature tRNA^Phe^ detected in this assay can be altered by the genetic background, normalization in this manner accounts for this variation. For example, in *mtr10Δ* and *ssa2Δ* strains approximately 50% and 30% more mature tRNA^Phe^, respectively, is detected as compared to WT cells (Supplementary Figure S2). Since similar findings are observed when using a 5′ exon or a 3′ exon probe, it is unlikely that this is due to an altered ability of the probe to hybridize due to the modification status of the tRNA. Rather, this could be due to an increase in mature tRNA^Phe^ production or stability.

In strains deficient in Mtr10, there is a >2-fold increase in levels of mature tRNA^Phe^ lacking the wybutosine modification as compared to WT cells, in both fed and amino acid-deprived cells (Figure 2A). As compared to fed WT cells, in which 92.8% of tRNA^Phe^ contains wybutosine, only 72.5% of tRNA^Phe^ from fed *mtr10Δ* cells contain the wybutosine modification (Supplementary Table S1). These findings are in agreement with previous reports indicating that Mtr10 functions in amino acid deprivation-induced tRNA retrograde nuclear import (18). Additionally, these results provide, for the first time, strong biochemical evidence of a constitutive role of Mtr10 in this process.

Next, we used the HCl/aniline assay to assess levels of tRNA^Phe-yW^ in *ssa2Δ* cells under fed and amino acid starvation conditions. Ssa2, a major Hsp70 protein, functions in amino acid deprivation-induced, but not constitutive, nuclear import of tRNAs (27). Accordingly, while levels of mature tRNA^Phe^ lacking yW are similar in WT and *ssa2Δ* cells treated sequentially with HCl and aniline under fed conditions, *ssa2Δ* cells deprived of amino acids for 2 h displayed a 30% increase in unmodified mature tRNA^Phe^ (Figure 2B). Together, the data show that the HCl/aniline assay is able to replicate previous findings that Mtr10 and Ssa2 are involved in the retrograde nuclear import process under conditions of amino acid deprivation, and that Mtr10 also functions as a constitutive importer. Thus, this assay can be used to identify gene products that mediate tRNA^Phe^ retrograde nuclear import and re-export under both constitutive and stress-induced conditions.

### Amino acid deprivation does not alter detectable levels of tRNA^Phe-yW^

**Figure 3.**
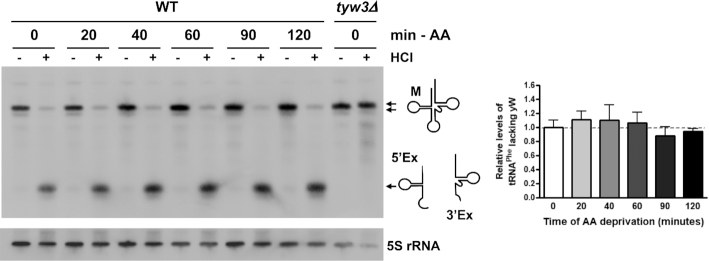
Amino acid deprivation does not alter levels of tRNA^Phe-yW^. Wild-type (WT) cells were grown in the absence of amino acids for 0–120 min. Small RNAs were isolated, treated with or without HCl, incubated with aniline, and visualized by Northern blot using the probe shown in Figure 1B. A representative Northern blot is shown. The *tyw3Δ* strain serves as a negative control and 5S rRNA levels serve as a loading control. The relative levels of tRNA^Phe^ lacking wybutosine (yW) following 0–120 min amino acid deprivation were calculated by dividing the amount of mature tRNA^Phe^ (M) in the HCl-treated sample by the total amount of tRNA^Phe^ (mature and cleaved) in that lane (*n* = 7 for 0 min; *n* = 2 for 20–90 min; *n* = 8 for 120 min). All data are normalized to WT in the presence of amino acids (set to 1) and are represented as mean ± SEM.

Many stress conditions are known to result in the accumulation of tRNAs in the nucleus, such as amino acid deprivation (18). A recent study examining the kinetics of tRNA import and re-export upon nutrient deprivation in mouse embryonic fibroblasts microinjected with fluorescently labeled tRNA, found that tRNAs accumulate in the nucleus upon nutrient deprivation and the rate constants of tRNA import and re-export both decrease as nutrient levels decrease (43). However, the rate of re-export decreases to a greater extent than the rate of import, accounting for the nuclear accumulation of tRNAs. Should the same be true in yeast, wild-type cells starved of nutrients would still be able to move tRNAs in and out of the nucleus, just at a reduced rate. In contrast, movement of tRNAs in cells that lack an importer or re-exporter would be inhibited. Therefore, we would expect that levels of tRNA^Phe^ containing the wybutosine modification would be minimally affected by amino acid starvation. Sequential HCl and aniline treatment of RNAs isolated from wild-type cells grown in the absence of amino acids for 20–120 min shows that similar to fed cells, mature tRNA^Phe^ cleavage is nearly complete (Figure 3). This indicates that wybutosine modification of tRNA^Phe^ is unaffected by decreased amino acid levels. Furthermore, these findings demonstrate the specificity of the HCl/aniline assay in the detection of importers and re-exporters, and not changes in import and re-export kinetics.

### Mex67 and Crm1, but not Los1 or Msn5, mediate constitutive re-export of mature tRNA^Phe^

**Figure 4.**
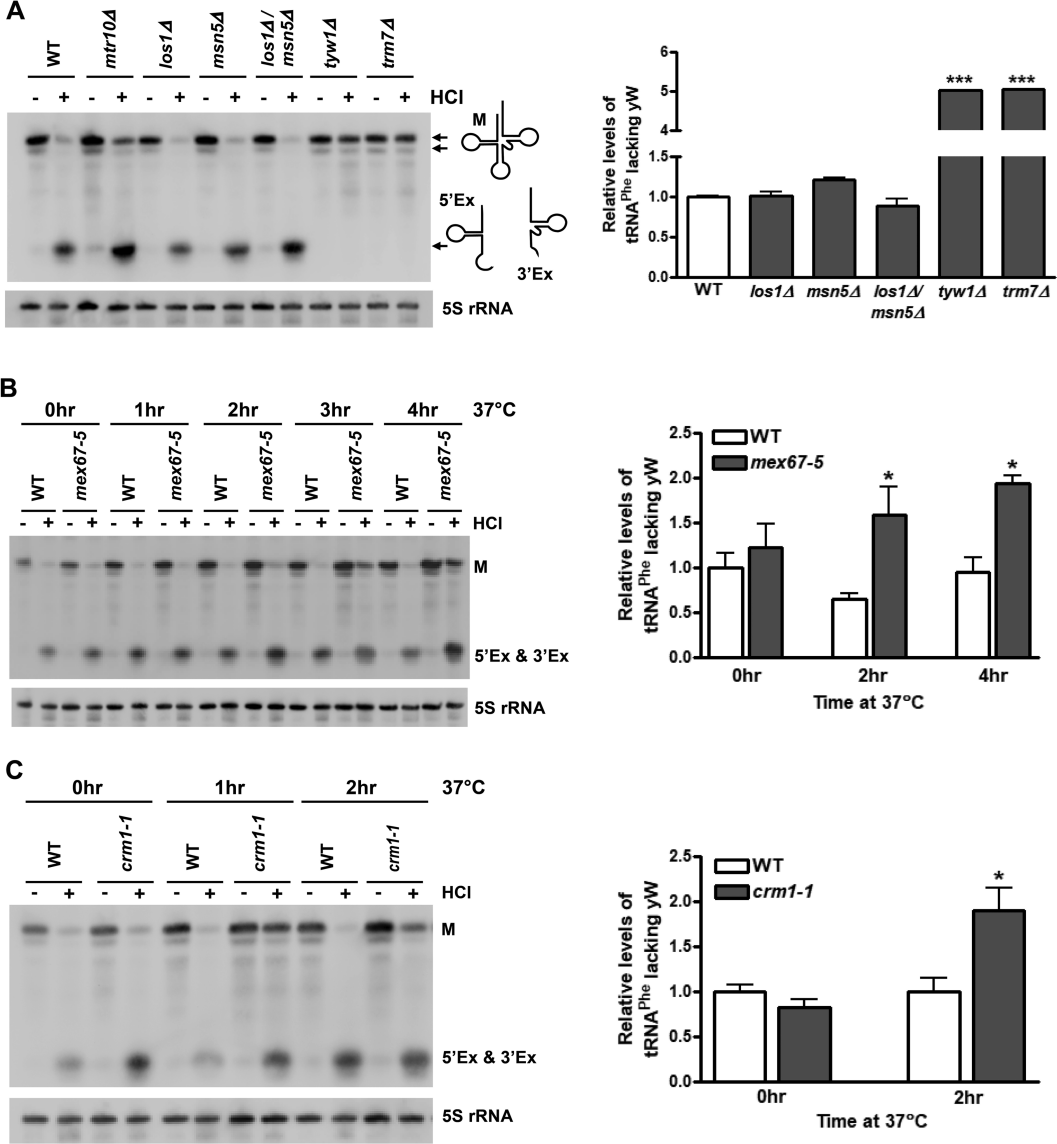
Mex67 and Crm1, but not the canonical tRNA exporters, Los1 or Msn5, mediate the re-export of tRNA^Phe^. (**A**) Small RNAs were isolated from wild-type (WT) or deletion strains, treated with or without HCl, incubated with aniline, and visualized by Northern blot using the probe shown in Figure 1B. A representative Northern blot is shown. 5S rRNA levels serve as a loading control. The relative levels of tRNA^Phe^ lacking wybutosine (yW) in each of the strains, shown in the graph on the right, are calculated by dividing the amount of mature tRNA^Phe^ (M) in the HCl-treated sample by the total amount of tRNA^Phe^ (mature and cleaved) in that lane (*n* = 5–6). All data was normalized to WT cells, which was set to 1. ***P < 0.001 relative to WT cells. (**B** and **C**) WT and *mex67-5* (*n* = 4) or *crm1-1* (*n* = 5–6) cells were grown at 23°C, then shifted to 37°C for the indicated time. Samples were prepared and analyzed as described in (A). All data are normalized to WT cells at 0 h, which is set to 1. *P < 0.05 relative to time-matched WT samples. All data are represented as mean ± SEM.

Next, we utilized the HCl/aniline assay to determine which, if any, of the known tRNA exporters function in the nuclear re-export of spliced tRNA^Phe^. Los1 and Msn5 are two canonical tRNA exporters. While Los1 functions in both tRNA primary export and re-export, Msn5 functions specifically in the latter step (10,42,44). Surprisingly, sequential HCl and aniline treatment of RNAs isolated from *los1Δ*, *msn5Δ* or *los1Δ msn5Δ* cells shows near complete cleavage of tRNA^Phe^, similar to wild-type cells (Figure 4A, left). Quantitation of the data indicates no statistically significant difference in the levels of tRNA^Phe^ lacking wybutosine in any of these strains relative to wild-type cells (Figure 4A, right). Together, these data indicate that neither Los1 nor Msn5 plays a substantial role in the constitutive re-export of tRNA^Phe^.

Recently, three additional proteins have been identified to function in the export of tRNAs. These proteins are Mex67 (29), Mtr2 (29) and Crm1 (28). We assessed whether each of these tRNA exporters function in the re-export of tRNA^Phe^ using the HCl/aniline assay. At the permissive temperature of 23°C, the temperature-sensitive *mex67-5* strain showed levels of HCl/aniline-induced cleavage of tRNA^Phe^ that were similar to wild-type cells (Figure 4B). However, upon shifting to the non-permissive temperature of 37°C for 2–4 h, this cleavage was reduced. Quantitation reveals approximately a 2-fold increase in the levels of tRNA^Phe^ lacking the wybutosine modification in *mex67-5* cells at these time points. As compared to WT cells grown at 37°C for 2 h, in which 92.6% of tRNA^Phe^ contains wybutosine, in *mex67-5* cells grown under the same conditions only 68.3% of tRNA^Phe^ contains the wybutosine modification (Supplementary Table S1). Supplementation of *mex67-5* cells with a high-copy plasmid containing the functional *MEX67* gene C-terminally tagged with protein A (29), partially rescues this phenotype (Supplementary Figure S3). Together, these data indicate that Mex67 functions in the constitutive re-export of tRNA^Phe^.

Cells have multiple parallel pathways for the subcellular trafficking of tRNAs between the nucleus and cytoplasm (6). In the absence of one pathway, another may compensate to ensure proper tRNA trafficking. Therefore, we assessed whether the canonical tRNA exporters, Los1 and Msn5, could function in the re-export of tRNA^Phe^ in cells deficient in Mex67. Surprisingly, even in the absence of a functional Mex67-mediated tRNA re-export pathway, neither overexpression of Los1 nor Msn5 rescued the re-export defect (Supplementary Figure S3). These findings further support the lack of function of these canonical tRNA exporters in the re-export of tRNA^Phe^.

Mex67 forms a heterodimer with Mtr2 to mediate the nuclear export of tRNAs (29) as well as mRNAs (45–47). Therefore, we assessed whether Mtr2 also plays a role in the re-export of tRNA^Phe^. Interestingly, upon sequential HCl and aniline treatment of RNAs isolated from temperature sensitive *mtr2* cells incubated at the non-permissive temperature of 37°C for up to 4 h, cleavage of tRNA^Phe^ was similar to wild-type cells (Supplementary Figure S4). These data suggest Mex67 may function independent of Mtr2 in the re-export of tRNA^Phe^. However, since the *mtr2* strain is less thermosensitive than *mex67-5* at 37°C (29), further studies are needed to confirm this finding.

A third protein involved in the nuclear export of tRNAs is Crm1 (28). To determine whether Crm1 functions in the re-export of tRNA^Phe^, RNAs were isolated from *crm1-1* cells grown at the non-permissive temperature of 37°C for up to 2 h and incubated sequentially with HCl and aniline. At the permissive temperature of 23°C, *crm1-1* cells showed levels of HCl/aniline-induced cleavage of tRNA^Phe^ similar to wild-type cells (Figure 4C). However, within 1 hr of exposure to the non-permissive temperature of 37°C, this cleavage was reduced. A two-fold increase in the levels of tRNA^Phe^ lacking wybutosine was observed after 2 h. These findings indicate that like Mex67, Crm1 also functions in the re-export of tRNA^Phe^.

## DISCUSSION

**Figure 5.**
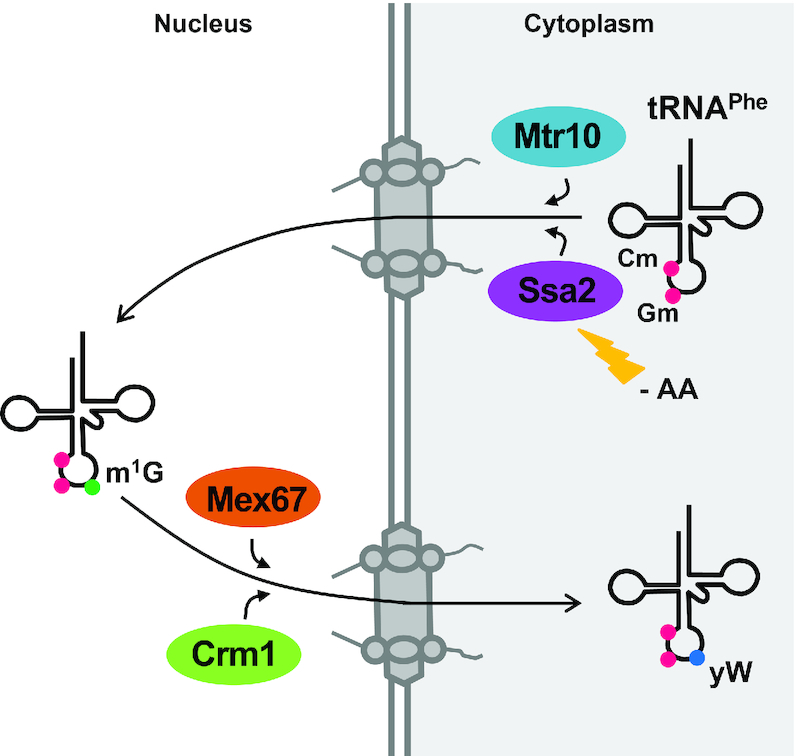
Model of the retrograde nuclear import and re-export pathways of tRNA^Phe^ in *S. cerevisiae*. Using the HCl/aniline assay, multiple proteins involved in the retrograde nuclear import and re-export of tRNA^Phe^ have been identified. Mtr10 functions in constitutive tRNA^Phe^ retrograde nuclear import, whereas Ssa2 is involved under conditions of amino acid deprivation. Both Mex67 and Crm1 mediate the constitutive re-export of tRNA^Phe^ to the cytoplasm. However, neither of the canonical tRNA exporters, Los1 nor Msn5, is required for this trafficking step.

In this study we demonstrate that the HCl/aniline assay is a valid and reliable test that can be used to identify constitutive and stress-induced tRNA retrograde nuclear importers and re-exporters in *S. cerevisiae*. This assay advances current methodologies to detect the wybutosine modification on tRNA^Phe^ and components of the retrograde trafficking pathway which previously relied on techniques such as LC/MS analysis (e.g. (20)). Unlike LC/MS which is labor intensive, technically challenging and requires costly instrumentation, the HCl/aniline assay is fast, easy, economical and does not require expensive instruments. Using the HCl/aniline assay, we provide the first biochemical evidence that Mtr10 functions in the constitutive import of tRNA^Phe^ and confirm a role for Ssa2 and Mtr10 in amino acid deprivation-induced import. Furthermore, we demonstrate that Mex67 and Crm1, but not the canonical exporters Los1 or Msn5, function in the nuclear re-export of tRNA^Phe^ (Figure 5).

### Constitutive and amino acid deprivation-induced tRNA^Phe^ retrograde nuclear import

It has long been known that tRNA^Phe^ requires trafficking via the retrograde nuclear import and re-export steps to become fully matured (6). However, the proteins mediating these movements were previously unknown. Through the use of the HCl/aniline assay, we now know that Mtr10 mediates the constitutive trafficking of tRNA^Phe^ into the nucleus. In support of previous findings (27,42), under conditions of amino acid deprivation, both Mtr10 and Ssa2 are involved in this process (Figure 5).

The findings that Mtr10 and Ssa2 are involved in the retrograde import of tRNA^Phe^ under conditions of amino acid deprivation are not surprising, given that these proteins have previously been identified in the nuclear import of other tRNA families. For example, unlike wild-type cells, *mtr10Δ* cells displayed a lack of nuclear accumulation of tRNA^Tyr^ and tRNA^His^ upon nutrient deprivation (18). Accordingly, using a Mtr10 shut-off strain Takano et al. (27) demonstrated reduced levels of tRNA^Pro^_UGG_ in the nucleus of cells deprived of amino acids. Ssa2 was similarly shown to play a role in the retrograde import of tRNAs under these conditions. Following two-hour amino acid deprivation, a lack of tRNA nuclear accumulation was observed in *ssa2Δ* cells, albeit at varying extents, for tRNA^Pro^_UGG_, tRNA_i_^Met^, tRNA^Lys^_CUU_, tRNA^Lys^_UUU_, and tRNA^Tyr^_GUA_ (27).

Import of tRNAs from the cytoplasm to the nucleus also occurs constitutively (42). However, it was previously unclear which proteins mediate this process. In agreement with our findings for tRNA^Phe^, Ssa2 does not play a role in the constitutive import of tRNA^Pro^_UGG_ or tRNA_i_^Met^ (27). However, genetic data suggested that Mtr10 may be involved in this process, given that unlike *msn5Δ* cells which show nuclear accumulation of tRNA^Tyr^, *msn5Δ mtr10Δ* cells do not accumulate tRNA^Tyr^ in the nucleus (42). This suggests Mtr10 functions upstream of the re-exporter Msn5. Using the HCl/aniline assay we were able to demonstrate a definitive role for Mtr10 in the constitutive import of tRNA^Phe^, as HCl/aniline-induced cleavage of tRNA^Phe^ was significantly inhibited in fed *mtr10Δ* cells. However, how Mtr10 mediates import of tRNAs into the nucleus remains unclear. The simplest model would be that Mtr10 binds directly to tRNAs to escort them into the nucleus, since it is a member of the β-importin family and is a nuclear importer of the mRNA nuclear export protein Npl3 (48) and the RNA subunit of telomerase TLC1 (49). Surprisingly, using co-immunoprecipitation assays followed by qPCR, a direct interaction between Mtr10 and several different tRNAs, including tRNA^Ile^, tRNA^Trp^, tRNA^Val^, and tRNA^Gln^, was not detected (44). However, co-immunoprecipitation of tRNA^Phe^ with Mtr10 was not assessed. Given these findings, it is likely that Mtr10 may not play a direct role in the import of tRNAs, but rather regulate the localization of other proteins that may be involved in the retrograde nuclear import process.

It also remains to be determined whether Mtr10 is the only pathway for constitutive import of tRNAs. Previous data on tRNA primary nuclear export (29) as well as findings from this and a previously published report (44) on the nuclear re-export step, show that different families of tRNAs are trafficked out of the nucleus by different pathways. Therefore, there may also be multiple parallel import pathways. The constitutive retrograde import process is required for maturation of tRNA^Phe^ (20) and tRNA quality control (22), at a minimum, suggesting that this pathway is essential to produce a full complement of mature tRNAs. Additionally, the changes in HCl/aniline-induced cleavage of tRNA^Phe^ in *mtr10Δ* or *ssa2Δ* cells (Figure 2; Supplementary Table S1) are modest compared to *tyw1-3Δ* or *trm7Δ* cells (Figure 4A, Supplementary Figure S1D). Together, this suggests there may be at least one additional constitutive pathway for the import of tRNAs back to the nucleus. A genome-wide screen using the HCl/aniline assay would be able to identify such proteins, as strains defective in these proteins would display a reduced ability to form tRNA^Phe-yW^.

### Re-export of tRNA^Phe^ by Mex67 and Crm1, but not the canonical tRNA exporters, Los1 or Msn5

Through the use of the HCl/aniline assay, re-export of tRNA^Phe^ was shown to be mediated by Mex67 and Crm1, but not Los1 or Msn5 (Figure 4). These results are surprising given that Los1 and Msn5 are known to be the canonical tRNA exporters, with Los1 mediating both the primary export and re-export steps, and Msn5 mediating the latter (10,11,42,44). These findings are even more surprising given that Los1 has been shown to mediate primary export of specifically tRNA^Phe^ in *S. cerevisiae* (29). Yet here, even in cells deficient in Mex67, overexpression of Los1 was unable to rescue the defect in tRNA^Phe^ re-export. These findings contradict biochemical and structural studies indicating that Los1 recognizes the tRNA backbone elbow and acceptor stem and not the intron or anticodon loop, and should thus be able to recognize all families of tRNAs (9,50). Perhaps Los1 is able to re-export tRNA^Phe^ in the absence of all other exporters or under specific cellular conditions. Even if so, it is clear that Los1 does not play a major role in the constitutive re-export of tRNA^Phe^.

In contrast to Los1, Msn5 preferentially interacts with spliced, aminoacylated tRNAs for their re-export from the nucleus by forming a quaternary complex with RanGTP, aminoacylated tRNA and Tef1/2 (44). It is currently unknown whether Msn5 interacts directly with aminoacylated tRNAs. Studies of the Msn5 homolog, exportin-5, predict that it interacts with the protruding 3′ end of the tRNA, much like it interacts with miRNAs in vertebrate cells (51). Since Tef1 also binds to this site, Msn5 may not interact with the tRNA directly, but with the Tef1/2-aminoacylated tRNA complex, and thus further studies of these interactions are required (44,52,53). It remains to be determined why tRNA^Phe^ is not a substrate for re-export by either Msn5 or Los1.

The re-export of tRNA^Phe^ to the cytoplasm is mediated by Mex67 and Crm1. The role of the former is perhaps not surprising given that the Mex67-Mtr2 mRNA export complex was recently identified to function in the primary export and re-export of intron-containing and spliced tRNA^Ile^, respectively, and overexpression of Mex67 in a *los1Δ* strain, but not a wild-type strain, was able to restore the primary tRNA^Phe^ export defect (29). Evidence for the requirement of Mtr2 heterodimerization with Mex67 in this process is lacking as our findings suggest Mex67-mediated re-export of tRNA^Phe^ may be Mtr2-independent. Although Mtr2 has been reported to function independent of Mex67 in the export of tRNAs in *T. brucei* (54), a role for Mex67 in tRNA re-export that is independent of Mtr2 has yet to be reported. There is precedence for Mex67-dependent, Mtr2-independent export of mRNA in humans, as the nuclear export of mRNA by TAP (the human homolog of Mex67) can act independently of p15 (the human homolog of Mtr2) (55). Mtr2, but not Mex67, is also able to independently associate with the nuclear pores in yeast (45). Further studies are needed to determine the role of Mtr2 in these processes.

In addition to Mex67, Crm1 was also shown to be involved in the re-export of tRNA^Phe^. Although the HCl/aniline assay cannot distinguish between gene products involved in retrograde import and re-export, the conclusion that Crm1 contributes to the re-export step, and not import, is based on three lines of evidence. First, Crm1 is known to export proteins that contain leucine-rich nuclear export signals, pre-ribosomes, mRNAs and telomerase RNA (56–59). Second, Crm1 was recently identified in a genome-wide screen to function in the export of intron-containing tRNA^Ile^, as well as tRNA^Tyr^ in yeast (28). Third, there are negative synthetic growth defects between *crm1-1* and *los1Δ* cells (28). Here, we identify an additional substrate for Crm1-mediated re-export. Since the interaction between Crm1 and RNAs is typically mediated by adaptor proteins (60), this is likely the case for Crm1-tRNA interaction as well. Although the adaptor proteins involved in Crm1-mediated tRNA re-export are not currently known, a genome-wide screen using the HCl/aniline assay would be able to identify such proteins.

### HCl/aniline assay limitations

Although quite powerful, it is important to note that this assay has several limitations, due in part to the remarkable complexities of the tRNA retrograde import and re-export processes. First, the HCl/aniline assay can only identify gene products involved in the nuclear import and re-export of tRNA^Phe^ since no other tRNAs in yeast carry the wybutosine modification. Therefore parallel trafficking pathways that are not used by tRNA^Phe^, but are used by other tRNA families, will go undetected. Previous work from our lab has demonstrated that the primary nuclear export of tRNAs is mediated by multiple parallel pathways, with different tRNA families utilizing different exporters. For example, upon incubation of *mex67-5* and *mtr2* cells at the nonpermissive temperature for 2 h, there was an accumulation of intron-containing pre-tRNAs for tRNA^Ile^_UAU_, tRNA^Tyr^_GUA_, tRNA^Trp^_CCA_ and tRNA^Pro^_UGG_, but not the other six intron-containing tRNA families. This shows that Mex67 and Mtr2 export some pre-tRNAs, but not others. In support of this finding, our work shows that the re-export of tRNA^Phe^ is mediated by Mex67 and Crm1, but not Los1 or Msn5. Conversely, other tRNAs, like tRNA^Ile^ and tRNA^Tyr^, are re-exported by Los1 and Msn5 (26,44). A similar phenomenon of tRNA selectivity was recently observed in the retrograde import process. In human cells, selective import of tRNA families was identified in response to hydrogen peroxide stress (25). Therefore, it is expected that multiple pathways are used for the import and re-export of the different tRNA families.

Another limitation is that this assay is unable to distinguish gene products involved in the retrograde import vs. re-export steps. Therefore another approach, such as fluorescence *in situ* hybridization (FISH), will be required to differentiate any hits identified by this assay. Our lab has previously utilized FISH to determine the subcellular localization of several tRNA families under a variety of conditions (18,28,42). A gene product involved in the tRNA retrograde nuclear import step will display cytoplasmic localization of mature tRNA^Phe^ in a strain deficient for that particular gene, whereas a gene product involved in nuclear re-export will show nuclear accumulation. In addition to differentiating positive hits as importers or re-exporters, FISH can also be used to assess the role of any confirmed gene products from the HCl/aniline assay in the import or re-export of tRNA families other than tRNA^Phe^. This will provide a more complete picture of the selectivity of importers and re-exporters for different tRNA families.

Thirdly, the HCl/aniline assay will identify gene products that are either directly or indirectly involved in the tRNA retrograde nuclear import and re-export steps. As a result, direct interactions between an identified gene product and tRNA^Phe^ will need to be determined by alternative approaches, such as coimmunoprecipitation assays (29,44). However, by casting a broader net, this assay will be beneficial in identifying gene products that regulate these currently elusive trafficking events.

### Conclusion and future perspectives

As demonstrated here, the HCl/aniline assay is a new and powerful tool that can be used to identify the role of known importers and exporters on the retrograde nuclear import and re-export of tRNA^Phe^ in yeast. However, this assay also has the potential to be utilized in a much broader scope. Given the simplicity of this assay, its likely ability to be multiplexed, and the availability of yeast deletion and temperature sensitive collections, this method can easily be scaled up to a genome-wide level. A genome-wide HCl/aniline assay screen would likely reveal not only gene products involved in the retrograde nuclear import and re-export steps, but currently unknown regulators of these processes. Additionally, since proteins like Mtr10 (44), Mex67 (60) and Crm1 (56) may mediate trafficking by interacting with the tRNA through adaptors, rather than directly, novel tRNA-interacting proteins will likely be discovered. The findings from such a genome-wide screen combined with the findings from our previous screen for genes involved in tRNA biogenesis prior to the retrograde nuclear import step (28), will provide a near complete picture of the gene products involved in each step of tRNA biology.

## DATA AVAILABILITY

All data to support the conclusions in this study have been made available either within the paper or in the supplemental data file.

## Supplementary Material

gkaa879_Supplemental_File

## References

[1] Phizicky E.M., Hopper A.K. tRNA biology charges to the front. Genes Dev. 2010; 24:1832–1860. 20810645 10.1101/gad.1956510 PMC2932967

[2] Kirchner S., Ignatova Z. Emerging roles of tRNA in adaptive translation, signalling dynamics and disease. Nat. Rev. Genet. 2015; 16:98–112. 25534324 10.1038/nrg3861

[3] Shaheen R., Abdel-Salam G.M., Guy M.P., Alomar R., Abdel-Hamid M.S., Afifi H.H., Ismail S.I., Emam B.A., Phizicky E.M., Alkuraya F.S. Mutation in WDR4 impairs tRNA m(7)G46 methylation and causes a distinct form of microcephalic primordial dwarfism. Genome Biol. 2015; 16:210. 26416026 10.1186/s13059-015-0779-x PMC4587777

[4] Guy M.P., Shaw M., Weiner C.L., Hobson L., Stark Z., Rose K., Kalscheuer V.M., Gecz J., Phizicky E.M. Defects in tRNA anticodon loop 2′-O-methylation are implicated in nonsyndromic X-linked intellectual disability due to mutations in FTSJ1. Hum. Mutat. 2015; 36:1176–1187. 26310293 10.1002/humu.22897 PMC4643400

[5] Hopper A.K., Phizicky E.M. tRNA transfers to the limelight. Genes Dev. 2003; 17:162–180. 12533506 10.1101/gad.1049103

[6] Chatterjee K., Nostramo R.T., Wan Y., Hopper A.K. tRNA dynamics between the nucleus, cytoplasm and mitochondrial surface: location, location, location. Biochim. Biophys. Acta Gene Regul. Mech. 2018; 1861:373–386. 29191733 10.1016/j.bbagrm.2017.11.007 PMC5882565

[7] Hopper A.K., Schultz L.D., Shapiro R.A. Processing of intervening sequences: a new yeast mutant which fails to excise intervening sequences from precursor tRNAs. Cell. 1980; 19:741–751. 7363329 10.1016/s0092-8674(80)80050-x

[8] Arts G.J., Fornerod M., Mattaj I.W. Identification of a nuclear export receptor for tRNA. Curr. Biol. 1998; 8:305–314. 9512417 10.1016/s0960-9822(98)70130-7

[9] Arts G.J., Kuersten S., Romby P., Ehresmann B., Mattaj I.W. The role of exportin-t in selective nuclear export of mature tRNAs. EMBO J. 1998; 17:7430–7441. 9857198 10.1093/emboj/17.24.7430 PMC1171087

[10] Hellmuth K., Lau D.M., Bischoff F.R., Kunzler M., Hurt E., Simos G. Yeast Los1p has properties of an exportin-like nucleocytoplasmic transport factor for tRNA. Mol. Cell. Biol. 1998; 18:6374–6386. 9774653 10.1128/mcb.18.11.6374 PMC109223

[11] Sarkar S., Hopper A.K. tRNA nuclear export in saccharomyces cerevisiae: in situ hybridization analysis. Mol. Biol. Cell. 1998; 9:3041–3055. 9802895 10.1091/mbc.9.11.3041 PMC25586

[12] Kutay U., Lipowsky G., Izaurralde E., Bischoff F.R., Schwarzmaier P., Hartmann E., Gorlich D. Identification of a tRNA-specific nuclear export receptor. Mol. Cell. 1998; 1:359–369. 9660920 10.1016/s1097-2765(00)80036-2

[13] Yoshihisa T., Ohshima C., Yunoki-Esaki K., Endo T. Cytoplasmic splicing of tRNA in Saccharomyces cerevisiae. Genes Cells. 2007; 12:285–297. 17352735 10.1111/j.1365-2443.2007.01056.x

[14] Yoshihisa T., Yunoki-Esaki K., Ohshima C., Tanaka N., Endo T. Possibility of cytoplasmic pre-tRNA splicing: the yeast tRNA splicing endonuclease mainly localizes on the mitochondria. Mol. Biol. Cell. 2003; 14:3266–3279. 12925762 10.1091/mbc.E02-11-0757 PMC181566

[15] Huh W.K., Falvo J.V., Gerke L.C., Carroll A.S., Howson R.W., Weissman J.S., O'Shea E.K. Global analysis of protein localization in budding yeast. Nature. 2003; 425:686–691. 14562095 10.1038/nature02026

[16] Wan Y., Hopper A.K. From powerhouse to processing plant: conserved roles of mitochondrial outer membrane proteins in tRNA splicing. Genes Dev. 2018; 32:1309–1314. 30228203 10.1101/gad.316257.118 PMC6169838

[17] Takano A., Endo T., Yoshihisa T. tRNA actively shuttles between the nucleus and cytosol in yeast. Science. 2005; 309:140–142. 15905365 10.1126/science.1113346

[18] Shaheen H.H., Hopper A.K. Retrograde movement of tRNAs from the cytoplasm to the nucleus in Saccharomyces cerevisiae. Proc. Natl. Acad. Sci. U.S.A. 2005; 102:11290–11295. 16040803 10.1073/pnas.0503836102 PMC1183567

[19] Zaitseva L., Myers R., Fassati A. tRNAs promote nuclear import of HIV-1 intracellular reverse transcription complexes. PLoS Biol. 2006; 4:e332. 17020411 10.1371/journal.pbio.0040332 PMC1584419

[20] Ohira T., Suzuki T. Retrograde nuclear import of tRNA precursors is required for modified base biogenesis in yeast. Proc. Natl. Acad. Sci. U.S.A. 2011; 108:10502–10507. 21670254 10.1073/pnas.1105645108 PMC3127885

[21] Kessler A.C., Kulkarni S.S., Paulines M.J., Rubio M.A.T., Limbach P.A., Paris Z., Alfonzo J.D. Retrograde nuclear transport from the cytoplasm is required for tRNA(Tyr) maturation in T. brucei. RNA Biol. 2018; 15:528–536. 28901827 10.1080/15476286.2017.1377878 PMC6103694

[22] Kramer E.B., Hopper A.K. Retrograde transfer RNA nuclear import provides a new level of tRNA quality control in Saccharomyces cerevisiae. Proc. Natl. Acad. Sci. U.S.A. 2013; 110:21042–21047. 24297920 10.1073/pnas.1316579110 PMC3876269

[23] Chu H.Y., Hopper A.K. Genome-wide investigation of the role of the tRNA nuclear-cytoplasmic trafficking pathway in regulation of the yeast Saccharomyces cerevisiae transcriptome and proteome. Mol. Cell. Biol. 2013; 33:4241–4254. 23979602 10.1128/MCB.00785-13 PMC3811888

[24] Chen D.F., Lin C., Wang H.L., Zhang L., Dai L., Jia S.N., Zhou R., Li R., Yang J.S., Yang F. et al. An La-related protein controls cell cycle arrest by nuclear retrograde transport of tRNAs during diapause formation in Artemia. BMC Biol. 2016; 14:16. 26941127 10.1186/s12915-016-0239-4 PMC4778291

[25] Schwenzer H., Juhling F., Chu A., Pallett L.J., Baumert T.F., Maini M., Fassati A. Oxidative stress triggers selective tRNA retrograde transport in human cells during the integrated stress response. Cell Rep. 2019; 26:3416–3428. 30893612 10.1016/j.celrep.2019.02.077 PMC6426654

[26] Whitney M.L., Hurto R.L., Shaheen H.H., Hopper A.K. Rapid and reversible nuclear accumulation of cytoplasmic tRNA in response to nutrient availability. Mol. Biol. Cell. 2007; 18:2678–2686. 17475781 10.1091/mbc.E07-01-0006 PMC1924813

[27] Takano A., Kajita T., Mochizuki M., Endo T., Yoshihisa T. Cytosolic Hsp70 and co-chaperones constitute a novel system for tRNA import into the nucleus. Elife. 2015; 4:e04659. 10.7554/eLife.04659 PMC4432389 25853343

[28] Wu J., Bao A., Chatterjee K., Wan Y., Hopper A.K. Genome-wide screen uncovers novel pathways for tRNA processing and nuclear-cytoplasmic dynamics. Genes Dev. 2015; 29:2633–2644. 26680305 10.1101/gad.269803.115 PMC4699390

[29] Chatterjee K., Majumder S., Wan Y., Shah V., Wu J., Huang H.Y., Hopper A.K. Sharing the load: Mex67-Mtr2 cofunctions with Los1 in primary tRNA nuclear export. Genes Dev. 2017; 31:2186–2198. 29212662 10.1101/gad.305904.117 PMC5749166

[30] Li Z., Vizeacoumar F.J., Bahr S., Li J., Warringer J., Vizeacoumar F.S., Min R., Vandersluis B., Bellay J., Devit M. et al. Systematic exploration of essential yeast gene function with temperature-sensitive mutants. Nat. Biotechnol. 2011; 29:361–367. 21441928 10.1038/nbt.1832 PMC3286520

[31] Jones G.M., Stalker J., Humphray S., West A., Cox T., Rogers J., Dunham I., Prelich G. A systematic library for comprehensive overexpression screens in Saccharomyces cerevisiae. Nat. Methods. 2008; 5:239–241. 18246075 10.1038/nmeth.1181

[32] Guy M.P., Podyma B.M., Preston M.A., Shaheen H.H., Krivos K.L., Limbach P.A., Hopper A.K., Phizicky E.M. Yeast Trm7 interacts with distinct proteins for critical modifications of the tRNAPhe anticodon loop. RNA. 2012; 18:1921–1933. 22912484 10.1261/rna.035287.112 PMC3446714

[33] Droogmans L., Grosjean H. Enzymatic conversion of guanosine 3′ adjacent to the anticodon of yeast tRNAPhe to N1-methylguanosine and the wye nucleoside: dependence on the anticodon sequence. EMBO J. 1987; 6:477–483. 3556165 10.1002/j.1460-2075.1987.tb04778.x PMC553419

[34] Jiang H.Q., Motorin Y., Jin Y.X., Grosjean H. Pleiotropic effects of intron removal on base modification pattern of yeast tRNAPhe: an in vitro study. Nucleic Acids Res. 1997; 25:2694–2701. 9207014 10.1093/nar/25.14.2694 PMC146816

[35] Pintard L., Lecointe F., Bujnicki J.M., Bonnerot C., Grosjean H., Lapeyre B. Trm7p catalyses the formation of two 2′-O-methylriboses in yeast tRNA anticodon loop. EMBO J. 2002; 21:1811–1820. 11927565 10.1093/emboj/21.7.1811 PMC125368

[36] Noma A., Kirino Y., Ikeuchi Y., Suzuki T. Biosynthesis of wybutosine, a hyper-modified nucleoside in eukaryotic phenylalanine tRNA. EMBO J. 2006; 25:2142–2154. 16642040 10.1038/sj.emboj.7601105 PMC1462984

[37] Thiebe R., Zachau H.G. A specific modification next to the anticodon of phenylalanine transfer ribonucleic acid. Eur. J. Biochem. 1968; 5:546–555. 5698615 10.1111/j.1432-1033.1968.tb00404.x

[38] Ladner J.E., Schweizer M.P. Effects of dilute HCl on yeast tRNAPhe and E. coli tRNA1fMet. Nucleic Acids Res. 1974; 1:183–192. 4606505 10.1093/nar/1.2.183 PMC343337

[39] Burrows C.J., Muller J.G. Oxidative nucleobase modifications leading to strand scission. Chem. Rev. 1998; 98:1109–1152. 11848927 10.1021/cr960421s

[40] Wu J., Huang H.Y., Hopper A.K. A rapid and sensitive non-radioactive method applicable for genome-wide analysis of Saccharomyces cerevisiae genes involved in small RNA biology. Yeast. 2013; 30:119–128. 23417998 10.1002/yea.2947 PMC3668450

[41] Chan P.P., Lowe T.M. GtRNAdb 2.0: an expanded database of transfer RNA genes identified in complete and draft genomes. Nucleic Acids Res. 2016; 44:D184–D189. 26673694 10.1093/nar/gkv1309 PMC4702915

[42] Murthi A., Shaheen H.H., Huang H.Y., Preston M.A., Lai T.P., Phizicky E.M., Hopper A.K. Regulation of tRNA bidirectional nuclear-cytoplasmic trafficking in Saccharomyces cerevisiae. Mol. Biol. Cell. 2010; 21:639–649. 20032305 10.1091/mbc.E09-07-0551 PMC2820427

[43] Dhakal R., Tong C., Anderson S., Kashina A.S., Cooperman B., Bau H.H. Dynamics of intracellular stress-induced tRNA trafficking. Nucleic Acids Res. 2019; 47:2002–2010. 30496477 10.1093/nar/gky1208 PMC6393242

[44] Huang H.Y., Hopper A.K. In vivo biochemical analyses reveal distinct roles of beta-importins and eEF1A in tRNA subcellular traffic. Genes Dev. 2015; 29:772–783. 25838545 10.1101/gad.258293.115 PMC4387718

[45] Santos-Rosa H., Moreno H., Simos G., Segref A., Fahrenkrog B., Pante N., Hurt E. Nuclear mRNA export requires complex formation between Mex67p and Mtr2p at the nuclear pores. Mol. Cell. Biol. 1998; 18:6826–6838. 9774696 10.1128/mcb.18.11.6826 PMC109266

[46] Segref A., Sharma K., Doye V., Hellwig A., Huber J., Luhrmann R., Hurt E. Mex67p, a novel factor for nuclear mRNA export, binds to both poly(A)+ RNA and nuclear pores. EMBO J. 1997; 16:3256–3271. 9214641 10.1093/emboj/16.11.3256 PMC1169942

[47] Katahira J., Strasser K., Podtelejnikov A., Mann M., Jung J.U., Hurt E. The Mex67p-mediated nuclear mRNA export pathway is conserved from yeast to human. EMBO J. 1999; 18:2593–2609. 10228171 10.1093/emboj/18.9.2593 PMC1171339

[48] Senger B., Simos G., Bischoff F.R., Podtelejnikov A., Mann M., Hurt E. Mtr10p functions as a nuclear import receptor for the mRNA-binding protein Npl3p. EMBO J. 1998; 17:2196–2207. 9545233 10.1093/emboj/17.8.2196 PMC1170564

[49] Ferrezuelo F., Steiner B., Aldea M., Futcher B. Biogenesis of yeast telomerase depends on the importin mtr10. Mol. Cell. Biol. 2002; 22:6046–6055. 12167699 10.1128/MCB.22.17.6046-6055.2002 PMC134008

[50] Cook A.G., Fukuhara N., Jinek M., Conti E. Structures of the tRNA export factor in the nuclear and cytosolic states. Nature. 2009; 461:60–65. 19680239 10.1038/nature08394

[51] Okada C., Yamashita E., Lee S.J., Shibata S., Katahira J., Nakagawa A., Yoneda Y., Tsukihara T. A high-resolution structure of the pre-microRNA nuclear export machinery. Science. 2009; 326:1275–1279. 19965479 10.1126/science.1178705

[52] Nissen P., Kjeldgaard M., Thirup S., Clark B.F., Nyborg J. The ternary complex of aminoacylated tRNA and EF-Tu-GTP. Recognition of a bond and a fold. Biochimie. 1996; 78:921–933. 9150869 10.1016/s0300-9084(97)86714-4

[53] Nissen P., Kjeldgaard M., Thirup S., Polekhina G., Reshetnikova L., Clark B.F., Nyborg J. Crystal structure of the ternary complex of Phe-tRNAPhe, EF-Tu, and a GTP analog. Science. 1995; 270:1464–1472. 7491491 10.1126/science.270.5241.1464

[54] Hegedusova E., Kulkarni S., Burgman B., Alfonzo J.D., Paris Z. The general mRNA exporters Mex67 and Mtr2 play distinct roles in nuclear export of tRNAs in Trypanosoma brucei. Nucleic Acids Res. 2019; 47:8620–8631. 31392978 10.1093/nar/gkz671 PMC6794378

[55] Braun I.C., Herold A., Rode M., Izaurralde E. Nuclear export of mRNA by TAP/NXF1 requires two nucleoporin-binding sites but not p15. Mol. Cell. Biol. 2002; 22:5405–5418. 12101235 10.1128/MCB.22.15.5405-5418.2002 PMC133933

[56] Fornerod M., Ohno M., Yoshida M., Mattaj I.W. CRM1 is an export receptor for leucine-rich nuclear export signals. Cell. 1997; 90:1051–1060. 9323133 10.1016/s0092-8674(00)80371-2

[57] Stade K., Ford C.S., Guthrie C., Weis K. Exportin 1 (Crm1p) is an essential nuclear export factor. Cell. 1997; 90:1041–1050. 9323132 10.1016/s0092-8674(00)80370-0

[58] Woolford J.L. Jr, Baserga S.J. Ribosome biogenesis in the yeast Saccharomyces cerevisiae. Genetics. 2013; 195:643–681. 24190922 10.1534/genetics.113.153197 PMC3813855

[59] Gallardo F., Olivier C., Dandjinou A.T., Wellinger R.J., Chartrand P. TLC1 RNA nucleo-cytoplasmic trafficking links telomerase biogenesis to its recruitment to telomeres. EMBO J. 2008; 27:748–757. 18273059 10.1038/emboj.2008.21 PMC2265757

[60] Kelly S.M., Corbett A.H. Messenger RNA export from the nucleus: a series of molecular wardrobe changes. Traffic. 2009; 10:1199–1208. 19552647 10.1111/j.1600-0854.2009.00944.x PMC3702165

