# Supplementary material for: A novel assay provides insight into tRNAPhe retrograde nuclear import and re-export in S. cerevisiae
Source: Nucleic Acids Res. 2020 Oct 19;48(20):11577–88. doi: 10.1093/nar/gkaa879 (PMC7672469; doi:10.1093/nar/gkaa879)
Supplement: gkaa879_Supplemental_File [file gkaa879_supplemental_file.pdf]

SUPPLEMENTAL INFORMATION

A Novel Assay Provides Insight into tRNA^{Phe} Retrograde Nuclear Import and Re-export in S. *cerevisiae*

Regina T. Nostramo and Anita K. Hopper

CONTENTS:

Supplemental Methods
Supplemental References
Supplemental Table
Supplemental Figures 1-4

SUPPLEMENTAL METHODS

Strains and Plasmids

The *mtr2* strain was obtained from the temperature-sensitive mutant collection, kindly provided by Dr. P. Heiter (The University of British Columbia), as described (1). The multicopy pRS426 plasmid expressing C-terminally protein A-tagged Mex67 was made as described previously (1). The Los1-MORF plasmid was obtained from the yeast ORF collection (3).

HCl/aniline assay

To induce wybutosine base excision, 10 µg of small RNA were incubated with 20 µl of 0.12 M HCl in a final volume of 50 µl dH_2O for 3 hrs at 37 °C. Following incubation, RNA/HCl samples were neutralized with 11.38 µl of 5 mM KOH. Controls lacking HCl contained only RNA in dH_20. Next, 12 µl of HCl-treated, neutralized RNA or HCl-untreated RNA was incubated with an equal volume of 0.5 M aniline, pH 4.5 at 60 °C for 20 min to induce chain scission. RNAs were precipitated at -80 °C overnight by adding: 450 µl dH_20, 47.5 µl 3M sodium acetate pH 5.2, 1410 µl cold 100% ethanol, and 1 µl GlycoBlue Coprecipitant (Invitrogen). RNA was centrifuged at 15,000 x g for 20 min, washed in 1 ml 70% ethanol, and dissolved in 20 µl dH_2O.

Northern probes

Mature $tRNA^{Phe}$ and the 5' cleaved halves of $tRNA^{Phe}$ were detected using a digoxigenin-labelled probe that hybridizes to 18 nts at the 5' end of the 5' exon. To detect the 3' cleaved halves, an 18 nt digoxigenin-labelled probe was used that hybridizes to the 3' exon of $tRNA^{Phe}$, with the 3' end of the probe complementary to the 3rd nt at the 5' end of the 3' exon. The sequences of all probes used in this manuscript are as follows:

$tRNA^{Phe}$ 5'/3' exon probe:	5' CGAACACAGGACCTCCAGATCTTCAGTCTGGCGCTCTCCC 3'
$tRNA^{Phe}$ 5' exon probe:	5' CAACTGAGCTAAGTCCGC 3'
$tRNA^{Phe}$ 3' exon probe:	5' TGCGAACTCTGTGGATCGAACACAGGACCT 3'
$tRNA^{Leu}_{CAA}$ 3' exon probe:	5' CTCTTGCATCTTACGATAC
5S rRNA probe:	5' GCACCTGAGTTTCGCGTATGGT 3'

SUPPLEMENTAL REFERENCES

1. Chatterjee, K., Majumder, S., Wan, Y., Shah, V., Wu, J., Huang, H.Y. and Hopper, A.K. (2017) Sharing the load: Mex67-Mtr2 cofunctions with Los1 in primary tRNA nuclear export. *Genes Dev*, **31**, 2186-2198.
2. Huang, H.Y. and Hopper, A.K. (2015) In vivo biochemical analyses reveal distinct roles of beta-importins and eEF1A in tRNA subcellular traffic. *Genes Dev*, **29**, 772-783.
3. Gelperin, D.M., White, M.A., Wilkinson, M.L., Kon, Y., Kung, L.A., Wise, K.J., Lopez-Hoyo, N., Jiang, L., Piccirillo, S., Yu, H. *et al.* (2005) Biochemical and genetic analysis of the yeast proteome with a movable ORF collection. *Genes Dev*, **19**, 2816-2826.

SUPPLEMENTAL TABLES

Strain (growth conditions, if not 23°C)	tRNA^{Phe} probe	% tRNA^{Phe} containing yW Mean (SEM)
BY4741	5′ Exon	92.8% (0.8%)
BY4741	3′ Exon	90.5% (1.8%)
tyw1Δ, *tyw3Δ*, or *trm7Δ*	5′ Exon	0% (0%)
tyw1Δ, *tyw3Δ*, or *trm7Δ*	3′ Exon	0% (0%)
mtr10Δ	5′ Exon	72.5% (3.3%)
BY4741 (37°C, 2hrs)	5′ Exon	92.6% (1.0%)
mex67-5 (37°C, 2hrs)	5′ Exon	68.3% (2.7%)

Supplemental Table 1. Percentage of tRNA^{Phe} containing wybutosine in cells of different genetic backgrounds, determined by the HCl/aniline assay. RNA from wild-type (BY4741) cells, gene deletion cells or temperature sensitive mutant cells were treated with HCl and aniline, then analyzed by Northern blot using either a 5′ exon or 3′ exon tRNA^{Phe} probe. Probe sequences are listed in the Supplemental Methods. The percentage of tRNA^{Phe} containing wybutosine was calculated by first dividing the amount of mature tRNA^{Phe} by the amount of mature plus cleaved tRNA^{Phe}. This value was then subtracted from 1 and multiplied by 100 to get the percentage of tRNA^{Phe} containing wybutosine. For RNA isolated from *tyw1Δ*, *tyw3Δ*, or *trm7Δ* cells, no cleavage product was observed following HCl/aniline treatment. Data are expressed as mean with the SEM in parentheses. n = 2-3 for all samples.

SUPPLEMENTAL FIGURES

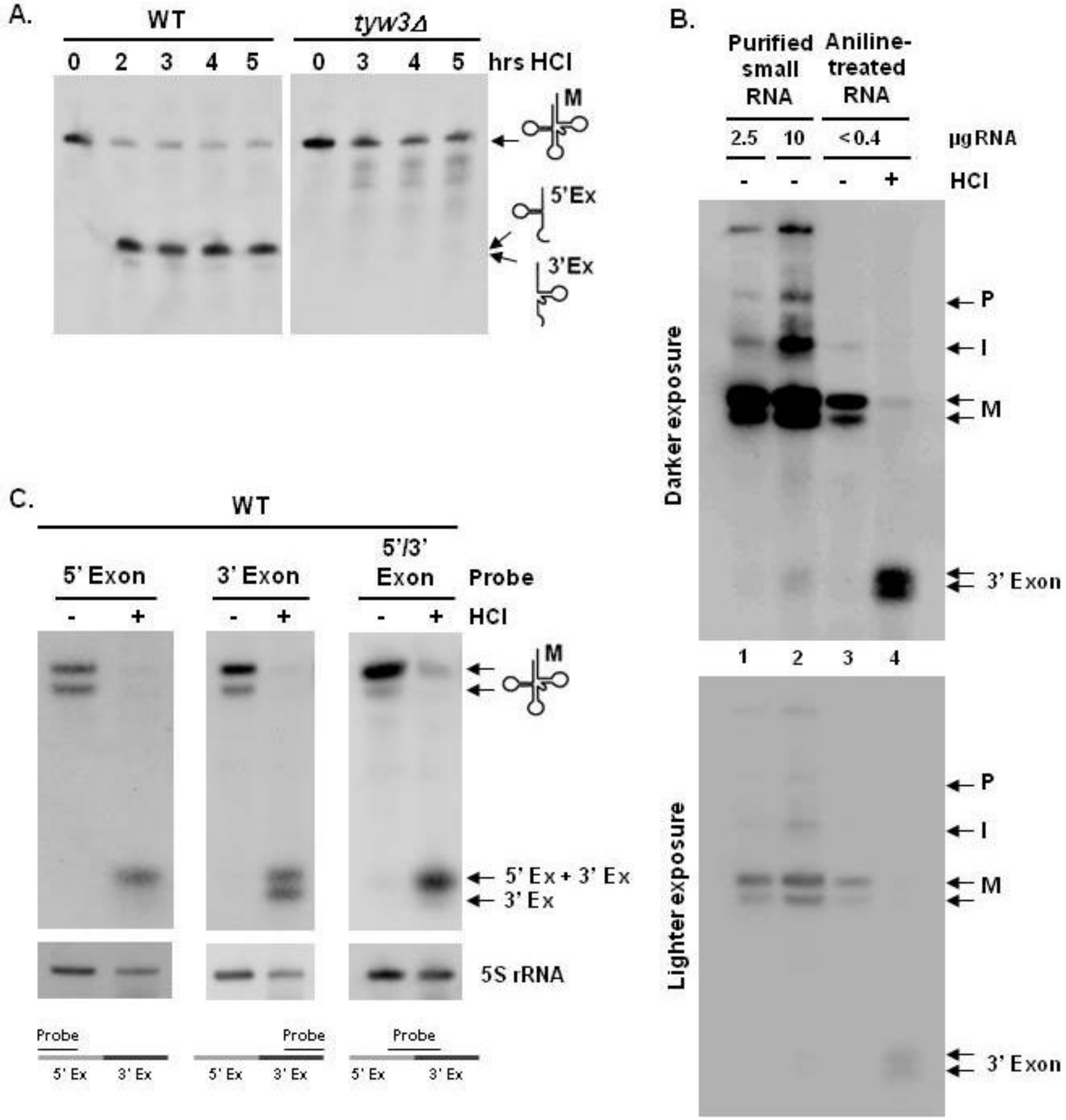

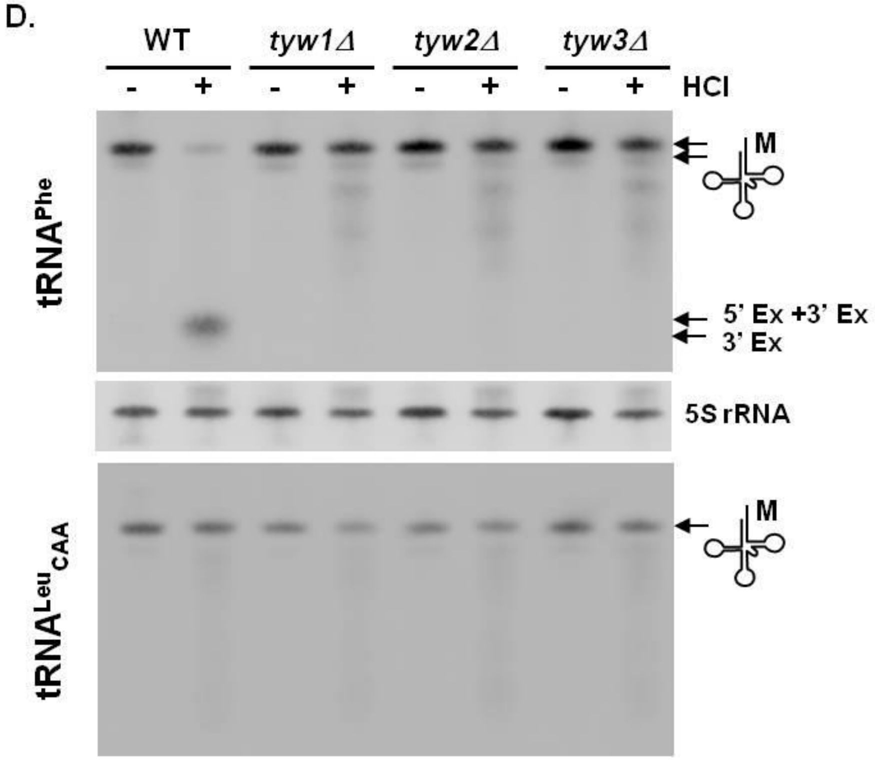

Supplemental Figure 1. Validation of the HCl/aniline assay to detect proteins involved in retrograde nuclear import as well as re-exporters of tRNA^{Phe}. A) Small RNAs isolated from wild-type (WT) or *tyw3Δ* cells were treated with HCl for 0-5 hrs and subsequently incubated with aniline. Northern blot analysis was performed using the probe described in Fig. 1B. The RNAs detected correspond to the mature (M), 5' exon (5'Ex) or 3' exon (3'Ex) of tRNA^{Phe}. B) Small RNAs were isolated from WT cells and the indicated amount of RNA was either left untreated (lanes 1 and 2), treated with aniline only (lane 3) or treated with HCl then aniline (lane 4). Northern blot analysis was performed using a probe that hybridizes to the 3' exon of tRNA^{Phe}. A darker (top) and lighter (bottom) exposure of the same blot are shown. P: primary tRNA^{Phe} containing 5' leader and 3' trailer; I: End-processed, intron-containing tRNA^{Phe}; M: Mature tRNA^{Phe}. C) Small RNAs isolated from WT cells were treated with or without HCl for 3 hrs and subsequently incubated with aniline. Northern blot analysis was performed using a 5' exon probe, a 3' exon probe or the exon junction probe described in (A). Levels of 5S rRNA serve as a loading control. D) Small RNAs were isolated from WT, *tyw1Δ*, *tyw2Δ* or *tyw3Δ* cells and treated and analyzed as in (C), using the tRNA^{Phe} exon junction probe (top) or a tRNA$^{Leu}_{CAA}$ 3' exon probe (bottom).

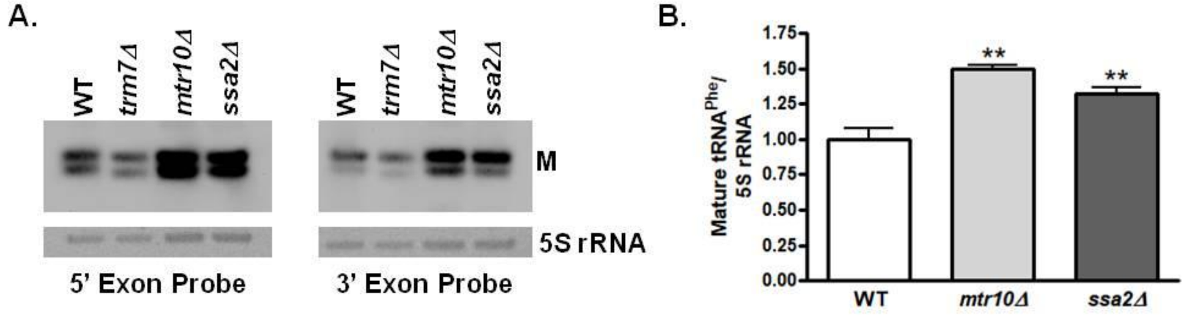

Supplemental Figure 2. tRNA^{Phe} levels are elevated in *mtr10Δ* and *ssa2Δ* strains. A) Northern blot of RNAs isolated from wild-type (WT), *trm7Δ*, *mtr10Δ* and *ssa2Δ* strains (n = 3). Blots were probed with an oligo that hybridizes to either the 5' exon (left) or 3' exon (right) of tRNA^{Phe}. 5S rRNA was used as a loading control. M: mature tRNA^{Phe}. B) Quantitation of mature tRNA^{Phe} levels from Northern blots for WT, *mtr10Δ* and *ssa2Δ* strains detected using a 3' exon probe. Mature tRNA^{Phe} levels are measured relative to 5S rRNA levels and all data are normalized to WT, which was set to 1. ** $p < 0.01$ relative to WT.

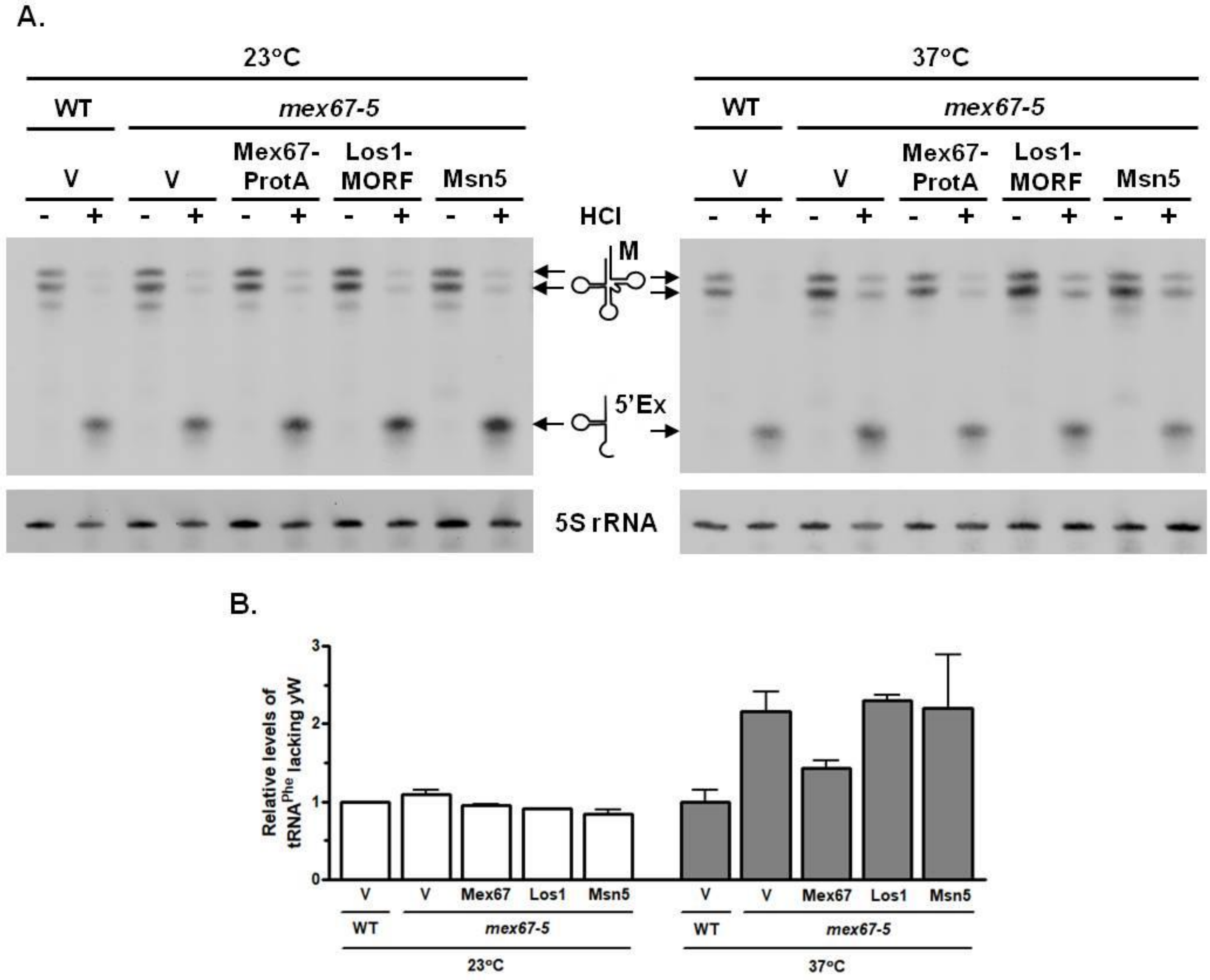

Supplemental Figure 3. Neither Los1 nor Msn5 overexpression rescues the tRNA^{Phe} re-export defect in *mex67-5* cells. A) Wild-type (WT) or *mex67-5* cells expressing either a multicopy vector (V) or the multicopy plasmid containing the functional *MEX67* gene C-terminally tagged with Protein A (Mex67-ProtA) (1), the functional *LOS1* gene C-terminally tagged with MORF (2), or the *MSN5* gene (untagged), were grown at 23 °C (left) or shifted to 37 °C for 2 hrs (right) (n = 2). Small RNAs were isolated and treated with or without HCl and subsequently incubated with aniline. Northern blot analysis was performed using a probe that hybridizes to the 5' exon. M: mature tRNA^{Phe}; 5'Ex: 5' exon of tRNA^{Phe}. 5S rRNA levels serve as a loading control. B) The relative levels of tRNA^{Phe} lacking wybutosine (yW) were calculated by dividing the amount of mature tRNA^{Phe} (M) in the HCl-treated sample by the total amount of tRNA^{Phe} (mature and cleaved) in that lane. Data are normalized to WT for the same temperature and WT is set to 1.

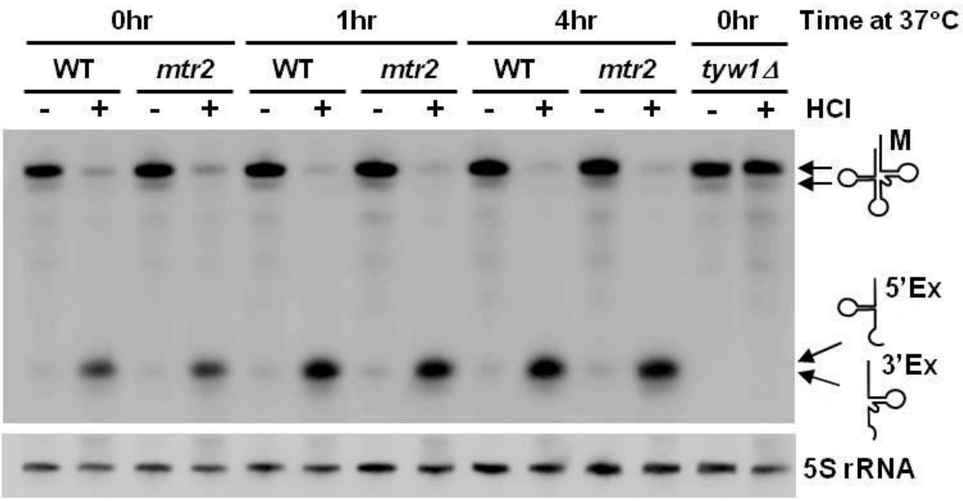

Supplemental Figure 4. Temperature-sensitive *mtr2* cells display no detectable defect in the re-export of tRNA^{Phe}. Wild-type (WT), *mtr2* and *tyw1Δ* cells (n = 2) were grown at 23°C or shifted to 37°C for the time indicated. Small RNAs were isolated and treated with or without HCl and subsequently incubated with aniline. Northern blot analysis was performed using the probe shown in Fig. 1B. 5S rRNA levels serve as a loading control.
